# Laparoscopic perineal hernia repair following pelvic exenteration: a case report

**DOI:** 10.1186/s12893-021-01237-9

**Published:** 2021-05-18

**Authors:** Méryl Dahan, David Krief, Nicolas Pouget, Roman Rouzier

**Affiliations:** 1 Département d’oncologie Chirurgicale, Institut Curie, PSL Research University, 35, Rue Dailly, 92210 Saint-Cloud, France; 2 Inserm U900, Cancer et génome : bioinformatique, biostatistiques et épidémiologie, Institut Curie, Saint Cloud, France; 3 Université Paris-Saclay, 78180 Montigny-le-Bretonneux, France

**Keywords:** Abdominoperineal excision, Case report, Laparoscopy, Prosthetic mesh, Perineal hernia

## Abstract

**Background:**

Acquired perineal hernia is a rare complication following extensive pelvic surgery. Radiotherapy is also a predisposing factor. Perineal hernia can cause chronic perineal pain, bowel obstruction, urinary disorders and a cosmetically disfiguring defect. The treatment of perineal hernia is surgical, usually consisting of mesh repair via an abdominal or perineal approach.

**Case presentation:**

We present a case report and a surgical video of a 42-year-old woman with history of a squamous cell carcinoma. This patient had 3 recurrences since the diagnosis and a symptomatic perineal hernia. Complete regression of the recurrent malignancy allowed us to treat the perineal hernia. We performed laparoscopic repair with prosthetic mesh in this patient who had undergone multiple surgeries and radiotherapy, while preserving the omental flap that was used to reconstruct the posterior part of the vagina.

**Conclusion:**

There is no consensus concerning the preferred surgical approach, perineal or laparoscopic, as no study has demonstrated the superiority of either of these approaches. Laparoscopic repair for an acquired perineal hernia is safe and feasible. However, further studies including randomized trials are required to precisely evaluate the best surgical approach and type of mesh.

**Supplementary Information:**

The online version contains supplementary material available at 10.1186/s12893-021-01237-9.

## Background

Acquired perineal hernia is a rare complication following extensive pelvic surgery with a reported incidence rate ranging between 0.6 and 7% after abdominoperineal resection or pelvic exenteration [1]. Perineal hernia is defined as protrusion of intraperitoneal organs via a defect in the pelvic floor and mainly occurs during the first postoperative year [2]. Perineal hernia can cause chronic perineal pain, bowel obstruction, urinary disorders and a cosmetically disfiguring defect [3]. However, perineal hernia is most commonly asymptomatic. The main identified predisposing factors are: female gender, smoking, immunosuppressive therapy, history of hysterectomy and history of pelvic chemoradiotherapy [4]. The diagnosis is based on clinical examination, confirmed by contrast-enhanced abdomen and pelvis CT scan and pelvic magnetic resonance imaging (MRI), as imaging visualizes the contents of the hernia and the condition of pelvic floor muscles prior to surgery [2]. The operative indication is based on the symptomatic nature of the hernia. The treatment of perineal hernia is surgical, usually consisting of mesh repair via an abdominal or perineal approach [1]. We report a case of laparoscopic mesh repair for perineal hernia in a patient with a history of pelvic exenteration. The laparoscopic approach was more appropriate in this patient, who had previously undergone multiple surgeries and perineal radiotherapy as well as omental flap repair to reconstruct the posterior part of the vagina. The perineal approach is the technique most commonly described in the literature, but did not appear to be suitable in our case because of the need to preserve the omental flap.

## Case presentation

**Fig. 1:**
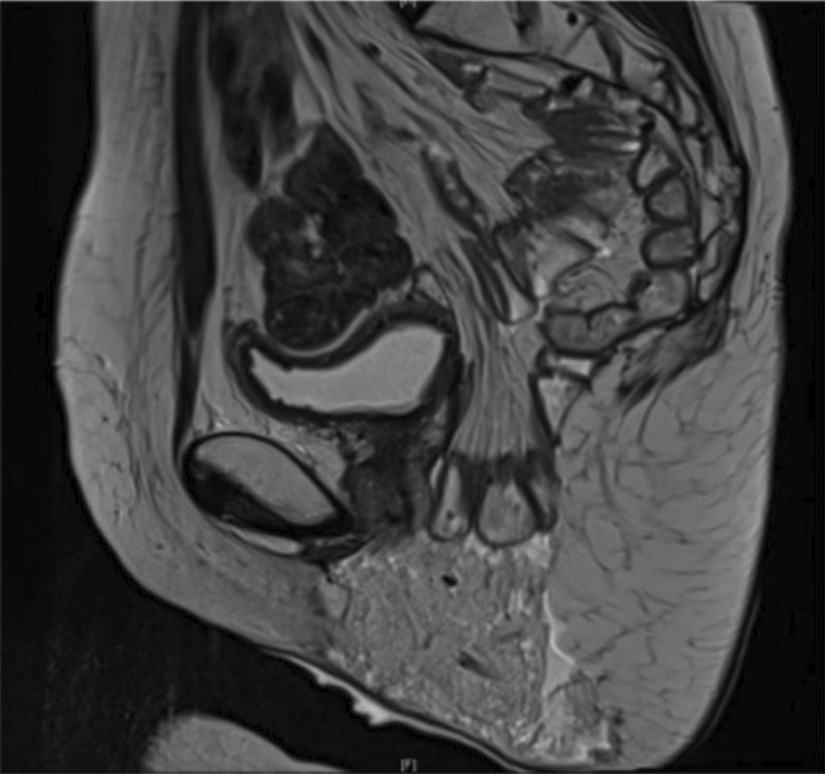
Sagittal pelvic MRI of the perineal hernia

We present a case report and a surgical video of a 42-year-old woman with perineal hernia. The manuscript has been written according to CARE guidelines. This patient presented with stage IIA squamous cell carcinoma of the cervix, initially treated by radiotherapy, chemotherapy and brachytherapy in December 2013. Surgery consisted of laparoscopic total hysterectomy and ovariectomy, together with pelvic and para-aortic lymphadenectomy. Early left paravaginal recurrence occurred in 2015 and was treated by resection of the left Bartholin gland. Another course of radiotherapy and chemotherapy was administered due to positive surgical margins (R1) of this resection. The patient presented another recurrence of her cervical carcinoma in February 2017 confirmed by pelvic CT scan and MRI. Due to the presence of a suspicious rectovaginal node, abdominoperineal resection with terminal colostomy was performed via a laparoscopic and perineal approach. We performed laparoscopic dissection of the left and right pararectal space to prepare an extralevator abdominoperineal excision and partial posterior colpectomy. We then performed radical posterior vulvectomy and a perianal incision at the lateral margin of the external anal sphincter and continued the dissection into the ischioanal fossa as far as the insertion of the pelvic floor muscles. We repaired the perineal defect and performed reconstruction of the posterior part of the vagina with an omental flap to preserve sexual function. After another course of chemotherapy and cycles of bevacizumab, the patient presented with her third recurrence two years later. Anterior pelvectomy was initially indicated due to a malignant mass infiltrating the right ischiopubic ramus and the right obturator internus muscle responsible for urethral retraction. This patient's case was discussed at a multidisciplinary consultation meeting and it was decided to treat her with chemotherapy. Complete regression of the recurrent malignancy allowed us to treat the perineal hernia (Fig. 1). The perineal hernia was causing pain and discomfort with a sensation of perineal heaviness. On physical examination, a reducible posterior perineal mass was palpated, which increased in volume during Valsalva manoeuvre.

### Laparoscopic repair

The patient was placed in the supine position. We performed open laparoscopy with the introduction of three 5-mm trocars under direct vision: suprapubic, right and left iliac fossa. Exploration did not reveal any signs of carcinoma. The patient was placed in the Trendelenburg position. No postoperative peritoneal adhesions were observed and the content of the sac was easily removed to visualize the omental flap that constituted most of the hernia sac. We pediculized the omental flap, that was vascularized by the left gastroepiploic artery and the Barkow’s arcade. The perineal hernia sac was not resected because of its extremely fibrous structure and the need to preserve the vaginal reconstruction. We introduced Symbotex prosthetic mesh, composed of two layers: one layer of 3D monofilament polyester for the abdominal wall, and the other hydrophilic collagen layer for the visceral side. We simply applied the mesh inside the pelvic cavity above the pediculized omental flap to restore the pelvic floor.

The mesh was then sutured:

- Posteriorly and laterally with Mersilene® 2.0 on the fibrous structure corresponding to the insertion of the previously resected left and right levator ani muscles.
- Superiorly with Vicryl® 2.0 to the umbilical fascia.

**Fig. 2:**
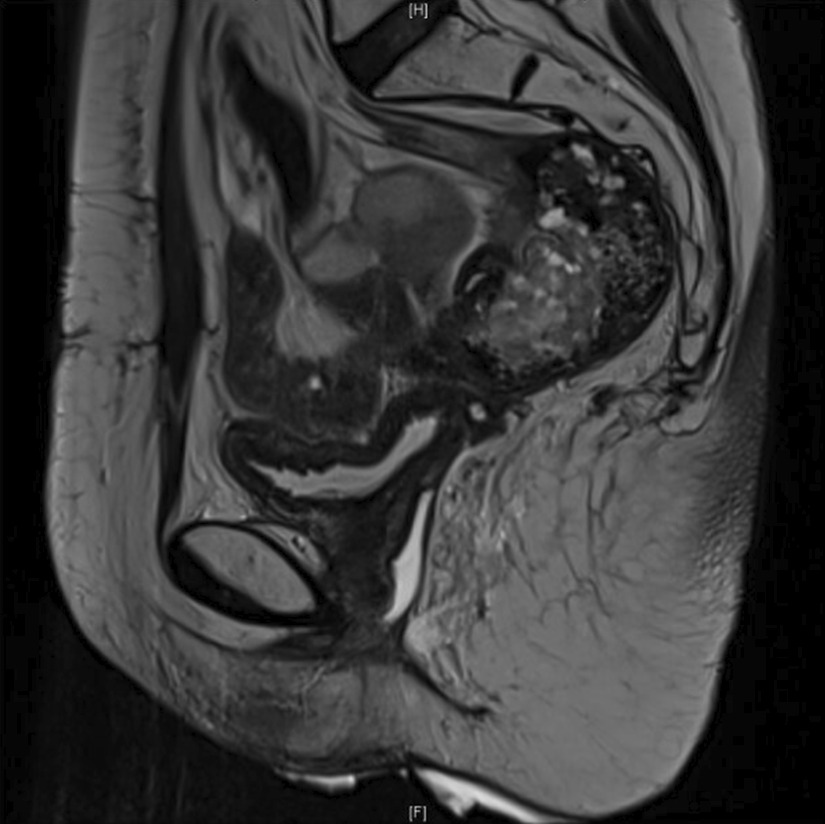
Sagittal pelvic MRI at 6 months follow-up

We did not immediately remove the excess skin. The immediate postoperative period was marked by urinary retention, requiring a bladder catheter for 5 days and urinary tract infection treated by antibiotics. Perineal pain was relieved and the patient was satisfied with the result. At 6-month follow-up, the patient remained asymptomatic with no perineal hernia recurrence on clinical examination and imaging (Fig. 2).

## Discussion and conclusion

Postoperative perineal hernia is a rare complication, first described in 1939 after proctectomy for rectal cancer [5]. However, this complication has been poorly studied, as most publications consist of case reports or small retrospective case series. No standardized surgical management of perineal hernia has therefore been defined. In 2012, Mjoli et al. systematically reviewed 43 cases of postoperative perineal hernia, including 22 patients treated via a perineal approach, 11 patients treated via an open abdominal approach, 3 patients treated via an open abdominoperineal approach, 2 patients treated via a laparoscopic-perineal approach and 5 patients treated by laparoscopy alone. These authors reported the superiority of mesh repair compared to non-mesh techniques in terms of recurrence [6]. There is no consensus concerning the preferred surgical approach, perineal or laparoscopic, as no study has demonstrated the superiority of either of these approaches [1]. In the largest review, published in 2017 (n = 108 cases), the perineal approach was used in almost 70% (n = 75) of cases, whereas laparoscopy was used in 23% (n = 25) of cases. An abdominal approach has also been occasionally used [7]. However, the laparoscopic approach presents several advantages. First, it allows better exposure for dissection of the contents of the hernia sac, hernial boundaries and pelvic contours [8]. It also provides good access for mesh positioning on solid structures, such as the sacrum and pelvic floor. Finally, omentoplasty can also be performed [2]. An omental flap is usually used to cover the pelvic defect. In our case, we initially performed an omental flap to cover the pelvic defect and for vaginal reconstruction in order to preserve sexual function. The main disadvantages of a laparoscopic approach remain the risk of adhesiolysis, which can be extensive following pelvic surgery, as well as a higher risk of mesh infection due to a colostomy or ileostomy in the operative field. Some studies have reported high recurrence rates after perineal hernia repair. A retrospective study of 24 perineal hernia repairs, published in 2019, reported lower recurrence and complication rates with a laparoscopic and abdominal approach (40%) compared to a perineal approach (50%) [9].

Few studies have specifically compared the type of mesh: biological or synthetic. In a recent retrospective study of 34 patients who underwent perineal repair, the use of biological mesh was associated with a 39% recurrence rate with a follow-up of 30 months compared to 31% with synthetic mesh, but this difference was not statistically significant (p = 0.642) [10]. Finally, no study has evaluated patient satisfaction and postoperative quality of life after perineal hernia repair. We used a two-sided mesh in our case: the 3D monofilament polyester side was placed in front of the bladder and the hydrophilic collagen side was placed in contact with the viscera. With this type of mesh, peritonization is unnecessary. The postoperative urinary retention observed in this patient was attributed to her history of multiple operations and irradiation.

Prophylactic mesh is increasingly used after extralevator abdominoperineal excision (ELAPE) or abdominoperineal resection (APR) to reduce the incidence of secondary perineal hernia [11. Prospective studies are necessary to evaluate this practice for gynaecological cancer, especially in the case of vaginal reconstruction with close proximity between the mesh and the flap that may increase the risk of postoperative infections.

In conclusion, laparoscopic repair for an acquired perineal hernia is safe and feasible. However, further studies including randomized trials are required to precisely evaluate the best surgical approach and type of mesh (Additional file 1).

## Supplementary Information

**Additional file 1**. Video of the perineal repair.

## Data Availability

The datasets used and analysed during the current study are available from the corresponding author on reasonable request.
